# P-374. Bacterial Outbreak Investigations Associated with Drug Diversion by U.S. Healthcare Personnel, 1990-2023

**DOI:** 10.1093/ofid/ofae631.575

**Published:** 2025-01-29

**Authors:** Rebecca Pierce, Axel A Vazquez Deida, Joe Perz, Kiran M Perkins

**Affiliations:** Centers for Disease Control and Prevention, Atlanta, Georgia; Centers for Disease Control and Prevention, Atlanta, Georgia; Centers for Disease Control and Prevention, Atlanta, Georgia; Centers for Disease Control and Prevention, Atlanta, Georgia

## Abstract

**Background:**

Diversion of injectable medications by healthcare personnel (HCP) is widely recognized as a source of bloodborne pathogen transmission. Drug diversion can also cause bloodstream infections (BSIs) but may not be identified as a transmission source due to the myriad of potential etiologies of bacterial outbreaks in healthcare settings. Here, we describe characteristics of drug diversion-related bacterial outbreaks to inform identification and control.

**Figure:**
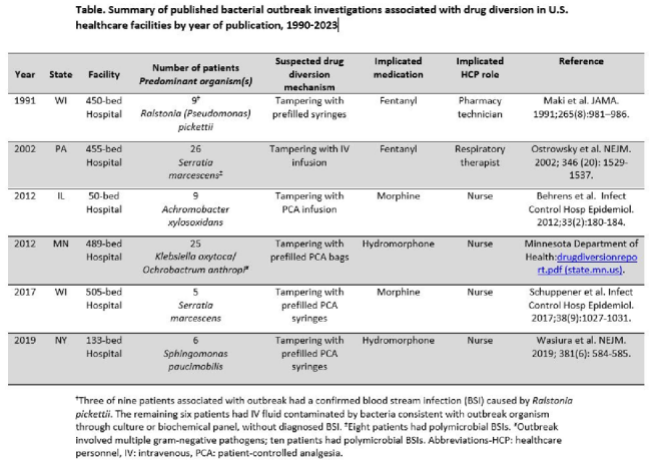

**Methods:**

We searched PubMed using the key terms “outbreak” or “cluster” and “diversion” to identify outbreaks reported from 1990-2023. Publication references were reviewed to identify additional reports. We excluded outbreaks outside the US and those caused by non-bacterial pathogens. We compiled patient- and outbreak-level data, including infection type, pathogen, length of stay, healthcare setting, diversion mechanism, and epidemiologic investigation strategies.

**Results:**

We identified six drug diversion-related bacterial outbreaks published between 1991-2019, involving 80 patients. 93% (n=74) of patients presented with BSIs; 93% (n=74) were identified after surgery or procedure; 24% (n=18) of patients with a BSI had a polymicrobial infection. Median outbreak duration was 119 days (range: 38-288). All outbreaks involved water-associated bacteria (Table) and four outbreaks reported BSI onset within 48 hours of admission for at least one patient. Outbreaks necessitated multiple epidemiologic strategies, including routine approaches (e.g., case-control study [n=4]) and techniques specific to medication tampering (e.g., medication concentration testing [n=4]). At least three patients (range: 3-24) in each outbreak had related isolates by molecular typing or whole genome sequencing. Four outbreaks reported tampering with opioids administered by patient-controlled analgesia (PCA) pumps.

**Conclusion:**

Certain features of healthcare BSI outbreaks, including involvement of water-associated bacteria, post-surgical populations, or rapid infection onset, should prompt consideration of drug diversion as a potential route of transmission. Multifaceted strategies, including evaluation for medication tampering, are needed to identify outbreaks from drug diversion.

**Disclosures:**

**All Authors**: No reported disclosures

